# The Long-Term Effects of Chronic Unpredictable Mild Stress Experienced During Adolescence Could Vary Depending on Biological Sex

**DOI:** 10.3390/ijms26031251

**Published:** 2025-01-31

**Authors:** Olesya M. Shirokova, Daria M. Kuzmina, Olga G. Zaborskaya, Natalia A. Shchelchkova, Elizaveta V. Kozliaeva, Svetlana A. Korotchenko, Vladimir I. Pershin, Petr I. Vasilchikov, Irina V. Mukhina

**Affiliations:** 1 Federal State Budgetary Educational Institution of Higher Education «Privolzhsky Research Medical University» of the Ministry of Health of the Russian Federation, 603005 Nizhny Novgorod, Russia; kuzmina_d@pimunn.net (D.M.K.); zaborskaya_o@pimunn.net (O.G.Z.); pershin_v@pimunn.net (V.I.P.); mukhina_i@pimunn.net (I.V.M.); 2 Institute of Biology and Biomedicine, Lobachevsky State University of Nizhny Novgorod, 23 Gagarina Ave., 602022 Nizhny Novgorod, Russia; eukarioshka@mail.ru; 3 Scientific Center of Genetics and Life Sciences, Sirius University of Science and Technology, Sirius Federal Territory, 354340 Krasnodar, Russia

**Keywords:** brain sexual dimorphism, chronic unpredictable mild stress, SigmaR1, behavior, immunohistochemistry

## Abstract

Sex differences in the neurobiology of responses to chronic stress have been widely discussed but remain poorly understood. We found that chronic unpredictable mild stress (CUMS) experienced during adolescence induced different behavioral patterns in adult males and females. Immunohistochemical analysis of the CA1 field of the dorsal and ventral hippocampus revealed no quantitative or morphological changes in astrocytes in the long term after CUMS. Real-time PCR analysis showed no increase in the expression level of SigmaR1 after CUMS relative to individual housekeeping genes. Analysis of mouse cerebral cortex homogenates showed that IL-1β levels only decreased after CUMS in males. However, the SigmaR1 levels were significantly higher in the CUMS groups than in the control groups in both sexes. It can be concluded that biological sex and age influence the response to CUMS, although not in all cases. Further studies are needed to understand the effects of chronic stress on males and females. This is important because men and women have different risks for stress and mental disorders.

## 1. Introduction

In modern biomedical science, psycho-emotional stress is considered a catalyst for various pathologies. The foundations for proper adaptations to stressors are formed in the family and educational environments. Therefore, particular attention is now being paid to the problem of stress in childhood and adolescence. Stress is a complex neuroimmunoendocrine reaction involving many organs and systems, with the immune, nervous, and endocrine systems being the main players in this process.

Chronic unpredictable mild stress (CUMS) has been shown to affect brain structure [1] and function [2]. Comprehensive approaches are needed to understand how early-life stress can reprogram cognitive and emotional brain networks, affect neurodevelopment, and increase the risk of psychopathology. These approaches must take into account the multifaceted nature of stress and its impact on nervous system development. When the body experiences both acute and chronic stress, the first system to respond is the hypothalamic–pituitary–adrenal (HPA) axis, which stimulates the adrenal cortex to synthesize and release cortisol (corticosterone in animals). Excess cortisol underlies many stress-related reactions [3]. In particular, high levels of circulating cortisol during chronic stress can cause long-term morphological changes in the hippocampus [4]. This part of the brain (for detailed information on its structure, see [5]) has an abundance of both mineralocorticoid and glucocorticoid receptors, so various stress-related psychopathologies affect learning and memory processes based on hippocampal activity [6], which is associated with behavioral responses to stress [7].

The behavioral changes induced by CUMS are also associated with dopaminergic hyperfunction and serotonergic hypofunction [8]. Interestingly, stress experienced in childhood leads to different behavioral strategies in males and females. This may be partly due to the different effects of sex steroids on HPA axis activity mediated by their effects on gene expression, protein synthesis, and cellular excitability via androgen and estrogen receptors [9]. It has also been shown that during puberty, sex steroid hormones, including progesterone, androgens, and estrogens, can further modulate brain development with long-term consequences for adult behavior [10]. Because sex steroids are involved in the regulation of behavioral and neuroendocrine responses to stress [11], they are able to modulate dopamine levels, with testosterone, for example, having a positive effect on dopamine levels and estrogens having the opposite effect. Dopamine levels are known to increase under acute stress and decrease under chronic stress, with the latter leading to maladaptive stress-related responses [12]. Indeed, there is normally marked sexual dimorphism in the function and structure of the dopaminergic system between males and females [13].

After CUMS, there are increases in the levels of dopamine, its metabolites, and tyrosine hydroxylase (TH) protein in the nucleus accumbens (NAc) [8]. Tyrosine hydroxylase plays a key role in the synthesis of catecholamines such as dopamine, norepinephrine, and epinephrine [13]. At the same time, unpredictable mild stress leads to decreased serotonin and tryptophan hydroxylase levels in the hippocampus and prefrontal cortex [8]. Changes in the prefrontal cortex affect higher cognitive functions such as decision-making, attention, and social behavior, while changes in the hippocampus affect the emotional state and memory. Thus, stress has opposing effects on different neurotransmitter systems in the brain. SigmaR1 knockout mice [14] show dysfunction in this system and signs of behavioral depression.

It is also well known that chronic stress and high cortisol levels suppress the immune response [7]. Chronic stress is assumed to increase the concentration of the pro-inflammatory cytokine IL-1 [15].

At the same time, melatonin acts as a homeostatic regulator of the HPA axis and is associated with a decrease in total corticosterone secretion [16]. However, this balance fails due to chronic-stress-induced cortisol resistance [15], which can lead to neuroinflammation. Unpredictable mild stress is known to increase inflammatory markers such as IL-6, IL-1β, and TNF-α and activate peripheral inflammatory pathways [17,18]. Emotional responses to stress may differ according to gender [19]. For example, women who react negatively to everyday stress have higher levels of inflammatory markers. Therefore, it is important to consider biological sex when investigating the mechanisms of exposure to unpredictable mild stress.

Furthermore, stress induces an immune system reaction that includes cytokine signaling and reliably leads to microglial responses in the hippocampus and prefrontal cortical regions [20], which in turn may lead to structural and functional changes in the brain following early-age CUMS.

However, mechanisms involving different types of proteins in the nervous system that contribute to the suppression of neuro-inflammatory responses and the polarization of microglia into the M1 type have been identified. For example, activation of the opioid receptor subtype triggers antidepressant, anti-inflammatory, and neuroprotective responses. This process is accomplished through the regulation of mitochondrial function and the interaction of the endoplasmic reticulum with mitochondria [21,22].

Alterations in the mitochondrial axis have been increasingly considered to underlie the pathogenesis of several diseases. Mitochondria are responsible for various types of signaling, including neuroinflammation and neurodegeneration [23]. A number of proteins of the outer mitochondrial membrane that affect mitochondrial dynamics and distribution by modulating fission–fusion mediators have been identified. These molecules include the mitofusin 1 (MFN1), MARCH5, and SigmaR1 proteins. MFN1 is an outer mitochondrial membrane protein required for mitochondrial fusion, MARCH5 is a mitochondrial E3 ubiquitin ligase, and SigmaR1 (one of two subtypes of sigma receptors) is a chaperone protein in the endoplasmic reticulum (ER) that modulates calcium signaling through the IP3 receptor [24].

Thus, chronic stress is considered a crucial factor in both behavioral changes and mitochondrial axis alterations, as well as in neuroinflammation and other functional changes in the central nervous system. Meanwhile, chronic stress during certain developmental periods may have different effects in males and females due to biological sex differences in cortisol levels and the HPA axis itself [7], different levels of sex steroids, and other factors. Taken together, this can lead to sex-specific responses to stress. However, the effects of early-life stressors in the context of biological sex remain poorly understood. Moreover, very little is known about how mild unpredictable stress during adolescence can affect mitochondrial signaling, inflammatory processes, and structural and functional features of the nervous system in general in female and male animals.

In this work, we assessed these parameters by modeling the consequences of CUMS in female and male C57BL/6 mice.

## 2. Results

### 2.1. Behavioral Phenotyping

**Figure 1:**
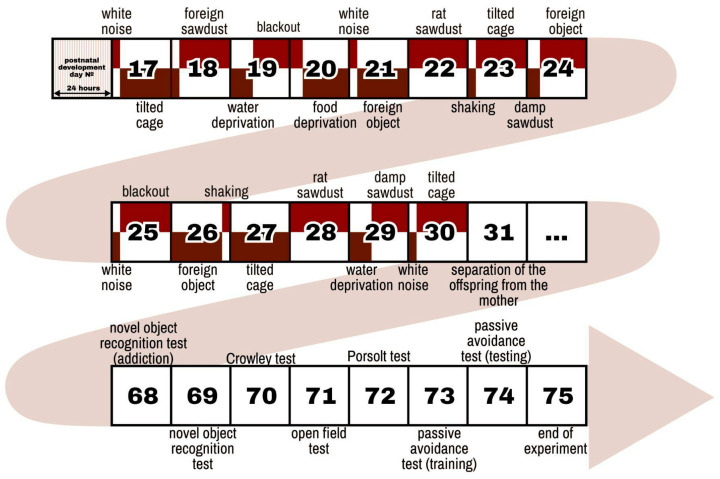
The Scheme scheme of the CUMS modelling modeling and behavioral test battery. CUMS modelling modeling was performed from on days 17 to 30 of the postnatal development of the mice (the detailed descriptions of the stressors is are presented in Table 1). On day 31 of postnatal development, the mice were separated from their mothers and placed in new cages with individuals of the same sex and age. The long-term effects of CUMS were assessed when the animals reached adulthood at 68–74 days of postnatal development. The behavioral tests were ordered from the least stressful to the most stressful tests.

The behavioral tests were ordered from the least stressful to the most stressful (Figure 1).

#### 2.1.1. Increased Anxiety in Females After Stressful Experiences

**Figure 2:**
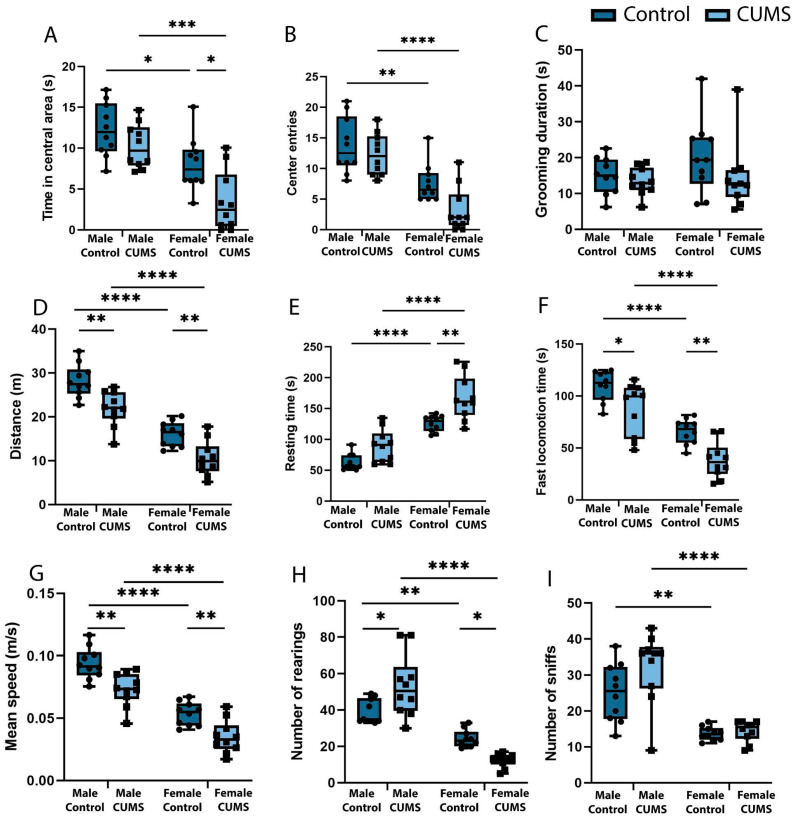
The effects of CUMS on male and female mice in the open-field test. (**A**) The time spent in the central area during the 5-min test. (**B**) The total number of entries into the central zone of the open-field arena. (**C**) The total duration of grooming acts during the test. (**D**) The distance traveled in the open field. (**E**) The total time spent resting. (**F**) The total duration of fast locomotion. (**G**) The mean walking speed of the mouse during the 5-min test. (**H**) The number of times the mouse stood vertically during the test. (**I**) The total number of sniffs during open-field exploration. The *p*-values were calculated using a two-way repeated-measures ANOVA (* *p* < 0.05; ** *p* < 0.01; *** *p* < 0.001; **** *p* < 0.0001).

Behavioral phenotyping showed that females subjected to CUMS exhibited higher levels of anxiety in an open-field test compared to control females and both groups of males and spent significantly less time in the central area of the arena than the control group (*p* = 0.068) (Figure 2A). The increased anxiety in females was also evidenced by a reduced number of entries into the central zone of the open-field arena (Figure 2B) compared to the control group and males subjected to CUMS. However, an increase in the duration of grooming, which is also a marker of anxiety, was not evident (Figure 2C).

#### 2.1.2. Decreased Locomotor Activity After Stress Is Characteristic of Both Sexes

The level of locomotor activity was significantly different between the mice subjected to CUMS and the control mice. The distance traveled (Figure 2D) was reduced in both the females subjected to CUMS (*p* = 0.0088) compared to the intact controls and the males subjected to CUMS (*p* = 0.0026) compared to the males that did not experience CUMS during early ontogeny. The females subjected to CUMS showed a significant increase in resting time (Figure 2E) compared to both the intact females (*p* = 0.0027) and the males subjected to CUMS (*p* < 0.0001). At the same time, the effect of stress was expressed as reductions in fast movements (Figure 2F) in the males (*p* = 0.0410) and females (*p* = 0.0081), as well as reductions in the average movement speed (Figure 2G) across the open-field arena in the females (*p* = 0.0088) and males (*p* = 0.0026).

#### 2.1.3. Differences in Exploratory Activity Between Males and Females

An analysis of the exploratory activity in the open-field test showed a statistically significant reduction in the number of rearing acts (Figure 2H) in the females subjected to CUMS (*p* = 0.0403) in the remote period after stress modeling. In contrast, the males subjected to CUMS showed an increase in exploratory activity, so the number of rearing acts was significantly higher in the experimental males (*p* = 0.0107) than in the control group. The total number of sniffs (Figure 2I) did not differ between the groups. At the same time, there was a tendency for this index of exploratory activity to increase in the males subjected to CUMS compared to both the control males and the control females.

#### 2.1.4. Decreased Hippocampus-Dependent Working Memory During Development of the Conditioned Passive Avoidance Reflex in Females and Decreased Hippocampus-Dependent Object Memory in Males After Stress

**Figure 3:**
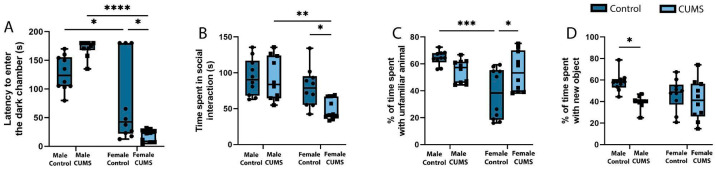
The effects of CUMS on learning and memory in the male and female mice. (**A**) The latency to enter the dark chamber in the passive avoidance test. (**B**) The social activity of the mice in the three-chamber Crowley test. (**C**) The proportion (percentage) of time spent with an unfamiliar animal in the three-chamber Crowley test. (**D**) The proportion (percentage) of time spent with a new object in the novel object recognition test. The *p*-values were calculated using a two-way repeated-measures ANOVA (* *p* < 0.05; ** *p* < 0.01; *** *p* < 0.001; **** *p* < 0.0001).

An examination of memory in the passive avoidance test (Figure 3A) revealed a decrease in working memory in the female CUMS group (*p* = 0.0143) but no significant differences in the males in the remote period after stress. A percentage analysis of animal learning in the novel object recognition test (Figure 3D) revealed that chronic stress experienced early in life caused a significant decrease in the time spent interacting with a novel object (*p* = 0.0134) in the males subjected to CUMS but not in the females.

#### 2.1.5. Increased Social Activity in Females After Stress

The social activity (Figure 3B) among the females increased dramatically in the CUMS group, as their time spent interacting with an unfamiliar animal in the first session of the three-chamber Crowley test was significantly higher than that of the control group (*p* = 0.0452). Moreover, the females from the experimental group showed a desire for social novelty (Figure 3C), which was expressed as a preference for an unfamiliar animal over a previously familiar one (*p* = 0.0326). At the same time, there were no significant changes among the males for this criterion. However, the group of males subjected to CUMS showed a tendency to decrease their social activity compared to the control group.

#### 2.1.6. CUMS Did Not Lead to the Development of Despair Behavior in the Remote Period

**Figure 4:**
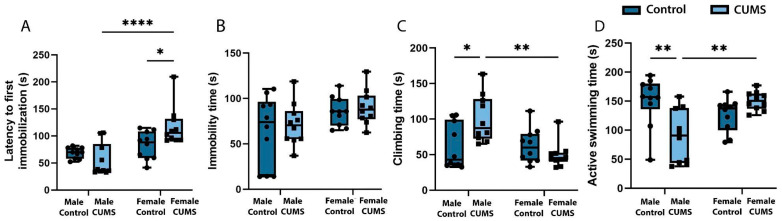
The depression-like behavior of the male and female mice in the Porsolt forced swim test after CUMS. (**A**) The time before the first immobilization. (**B**) The duration of immobilization. (**C**) The total climbing time. (**D**) The time spent actively swimming. The *p*-values were calculated using a two-way repeated-measures ANOVA (* *p* < 0.05; ** *p* < 0.01; **** *p* < 0.0001).

When analyzing the development of CUMS-induced depression-like behavior in the Porsolt forced swim test, it was found (Figure 4A) that the time to first immobilization was significantly longer in the female CUMS group than in the control group (*p* = 0.0436). The males subjected to CUMS had significantly less active swimming time (Figure 4D) (*p* = 0.0026) and significantly more climbing time (Figure 4C) (*p* = 0.0171) compared to the intact males and the females subjected to CUMS. At the same time, no significant changes in immobility time were found in any group (Figure 4B).

The behavioral tests demonstrated quite significant behavioral differences between the males and females, particularly in their learning ability, social behavior, and the emotional component of behavior. Memory and reflex development are mainly mediated by the dorsal hippocampus, whereas social activity in mice is mainly mediated by the ventral hippocampal CA1 [25] and CA2 regions [26] as well as their projections to other brain regions such as the NAc and the medial prefrontal cortex [27]. Interestingly, inflammation develops faster in the dorsal region of the hippocampus, while corticosterone accumulates faster in the ventral region [28]. We therefore decided to perform a general morphometric analysis of the dorsal and ventral hippocampal CA1 regions.

### 2.2. Immunohistochemical Study

#### 2.2.1. The Intensity of the MAP2 Staining in the Entorhinal Cortex and the Hippocampus Did Not Correlate with the Behavioral Phenotype

**Figure 5:**
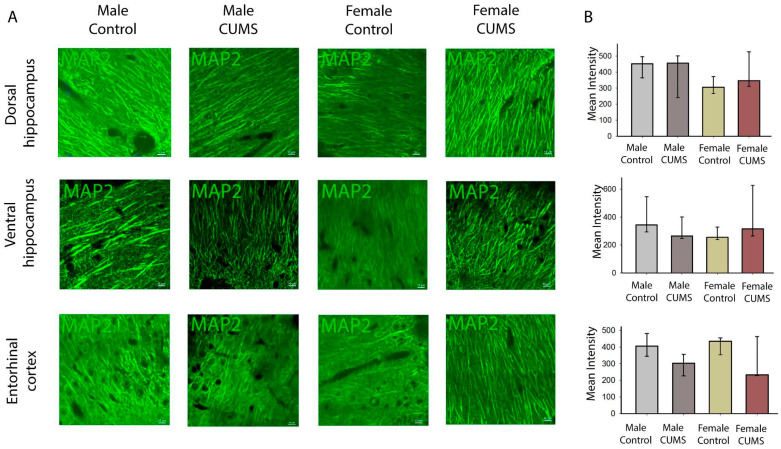
Immunofluorescence analysis of hippocampal (dorsal and ventral) and entorhinal cortex sections from the experimental and control mice. (**A**) Representative images of MAP2 labeling in the CA1 stratum radiatum of the dorsal and ventral hippocampus and in the entorhinal cortex. Scale bar: 10 µm. (**B**) The mean intensity of the MAP2 immunofluorescence in the different groups of mice.

MAP2 immunostaining is effective in detecting and assessing brain damage in cases of acute ischemia [29] and synaptic rearrangement [30]. Based on the results of a MAP2 fluorescence intensity analysis, no statistically significant differences between the experimental and control mice were found in the CA1 field of the dorsal and ventral hippocampus or in the entorhinal cortex (Figure 5). It appears that the effects observed in the behavioral phenotype are not related to a rearrangement of the cytoskeleton of dendritic outgrowths.

#### 2.2.2. Early-Age CUMS Did Not Induce Quantitative or Morphological Changes in Astrocytes in the Dorsal and Ventral Hippocampal CA1 Regions

**Figure 6:**
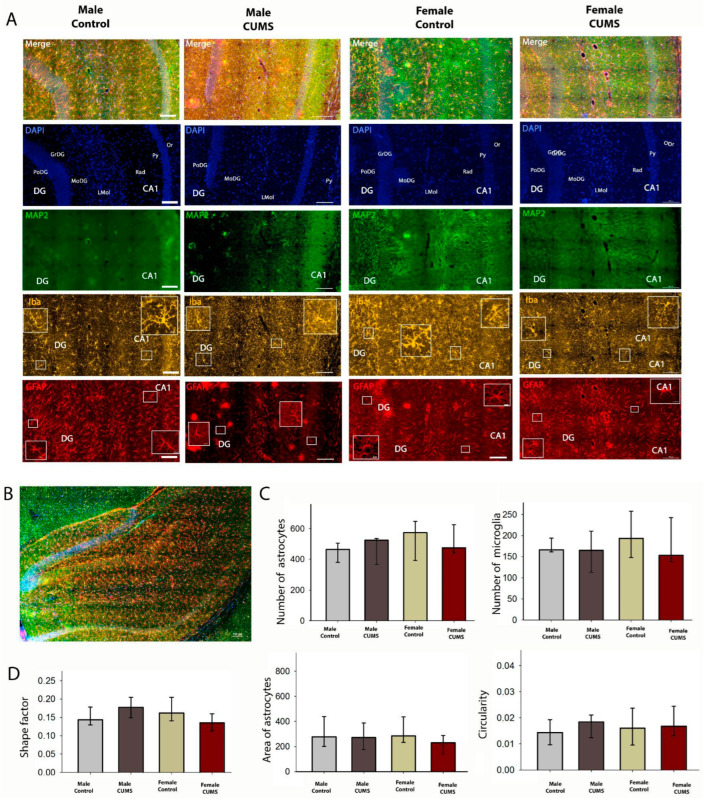
Immunofluorescence analysis of the dorsal hippocampus. (**A**) Triple labeling for MAP2, Iba1, and GFAP in the dorsal hippocampus and DAPI staining of all cell nuclei (blue). (**B**) A representative image of the dorsal hippocampus in a frontal slice of a mouse brain. (**C**) The numbers of astrocytes (GFAP-positive) and microglial cells (Iba-positive). (**D**) Morphometric analysis of the astrocytes in the CA1 field of the hippocampus using their shape factor, area, and Circularity. Scale bar: 100 µm.

**Figure 7:**
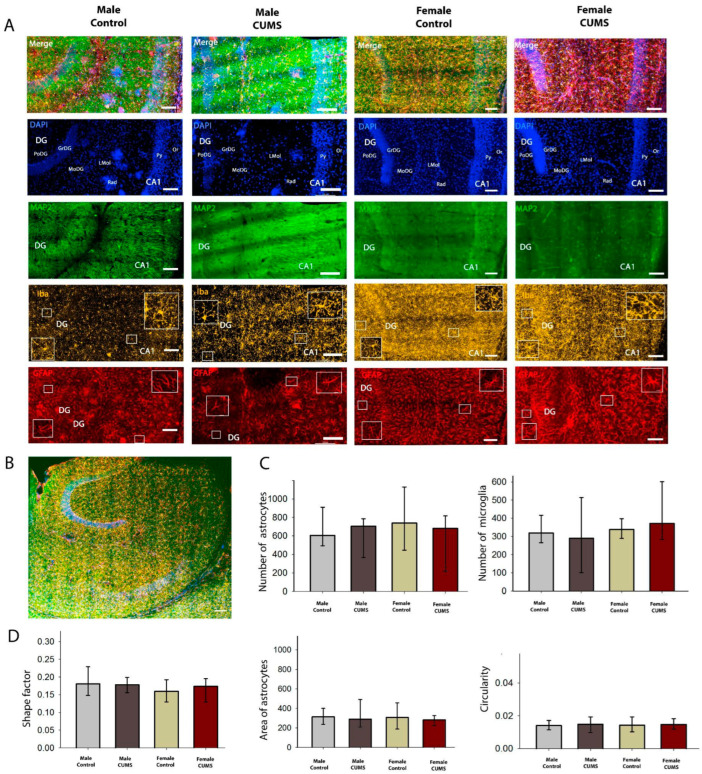
Immunofluorescence analysis of the ventral hippocampus. (**A**) Triple labeling for MAP2, Iba1, and GFAP in the ventral hippocampus. (**B**) A representative image of the ventral hippocampus in a horizontal slice of a mouse brain. (**C**) The numbers of astrocytes (GFAP-positive) and microglial cells (Iba-positive). (**D**) Morphometric analysis of the astrocytes in the CA1 field of the hippocampus using their shape factor, area, and Circularity. Scale bar: 100 µm.

Microglial and astrocytic cells in the CA1 field of the dorsal (Figure 6) and ventral (Figure 7) hippocampus were analyzed quantitatively. Although the number of glial cells in the CA1 stratum radiatum was significantly higher in the ventral hippocampus, the number of astroglial and microglial cells per mm^2^ did not change in the different experimental groups. The distribution of microglia within the hippocampal layers was uniform in all experimental groups, and no sexual dimorphism was found.

### 2.3. Enzyme Immunoassay of Cortex Homogenates

#### 2.3.1. CUMS Induced a Decrease in the IL-1β Level in the Cortical Homogenate from the Males

**Figure 8:**
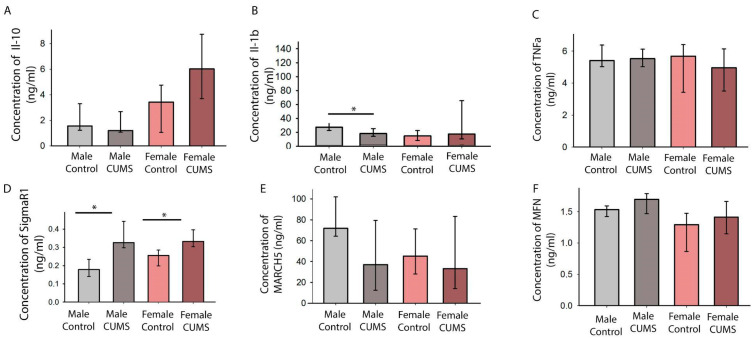
The levels of proteins of interest in the cerebral cortices of mice from different groups. (**A**) The concentration of the pro-inflammatory protein IL-10. (**B**) The concentration of the anti-inflammatory protein IL-1β. (**C**) The concentration of the pro-inflammatory protein TNFα. (**D**) The concentration of SigmaR1. (**E**,**F**) The concentrations of the mitochondrial proteins MARCH5 and MFN (* *p* < 0.05).

The IL-1β levels in the cortical homogenates only decreased after stress modeling in the males. No statistically significant differences between the groups were found when analyzing IL-10 and TNFα (Figure 8).

#### 2.3.2. CUMS Caused an Increase in the Hippocampal SigmaR1 Protein Level Regardless of Biological Sex

The females and males in the CUMS groups had significantly higher levels of SigmaR1 in their cortical homogenates than the control females (*p* = 0.016) and males (*p* = 0.004), respectively (Figure 8D). Thus, biological sex did not influence these dynamics, but it is worth noting that the difference between the males subjected to CUMS and the control males was more pronounced.

### 2.4. Real-Time PCR of Hippocampal Homogenates

#### CUMS Did Not Induce Changes in Hippocampal SigmaR1 or IL6 Gene Expression Regardless of Biological Sex

**Figure 9:**
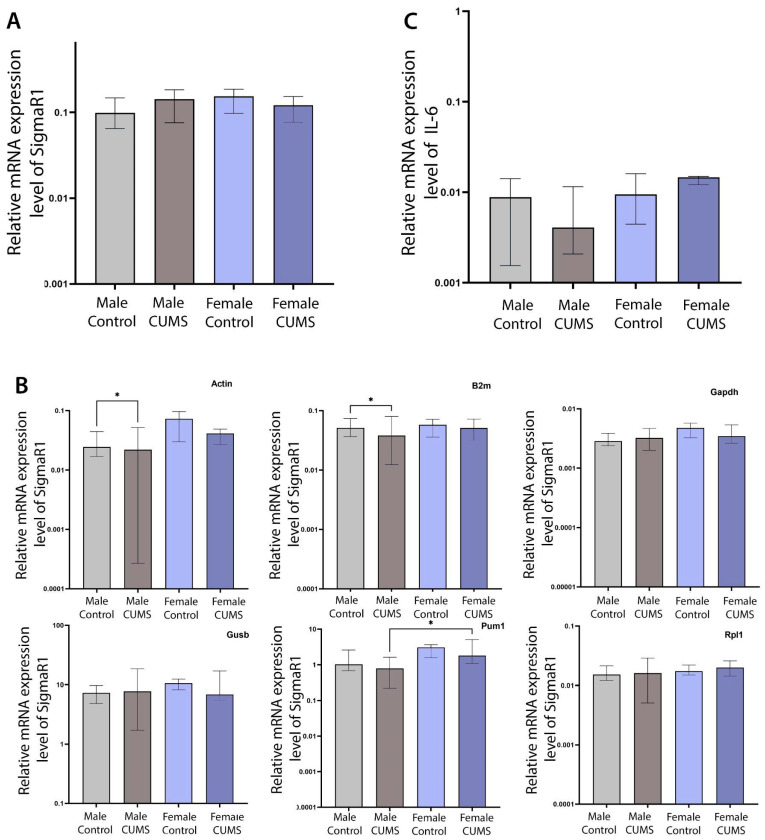
The relative expression of SigmaR1 and IL-6 in the hippocampus. (**A**) The expression level of SigmaR1 relative to 6 housekeeping genes. (**B**) The expression level of SigmaR1 relative to each housekeeping gene (ActB, B2m, Gapdh, Gusb, Pum1, and Rpl19). (**C**) The expression level of IL-6 relative to 6 housekeeping genes (* *p* < 0.05).

Real-time PCR showed no statistically significant changes in SigmaR1 gene expression relative to the geometric mean of all housekeeping genes used (ActB, B2m, Gapdh, Gusb, Pum1, and Rpl19) (Figure 9A). However, statistically significant differences were found (1) between the control males and the males exposed to CUMS when ActB was used as a housekeeping gene (an 11% decrease in SigmaR1 expression, *p* = 0.0483); (2) between the control males and the males exposed to CUMS when B2m was used as a housekeeping gene (a 35% decrease in SigmaR1 expression, *p* = 0.0453); and (3) between the males and females exposed to CUMS when Pum1 was used as a housekeeping gene (a 129% increase in SigmaR1 expression, *p* = 0.0197). No statistically significant differences were found between the groups when Gapdh, Rpl19, and Gusb were used individually as housekeeping genes (Figure 9B).

Real-time PCR showed no significant changes in IL-6 gene expression relative to the geometric mean of all housekeeping genes used (ActB, B2m, Gapdh, Gusb, Pum1, and Rpl19) (Figure 9C) and the individual genes (not presented here).

## 3. Discussion

Functional and structural characteristics of the brain associated with biological sex and age may influence responses to stress-related events early in life, but the neural networks involved are not fully understood. However, the existence of such sexual dimorphism is critical for both fundamental science and medicine, as men and women are known to have different risks of psychopathology [31].

Indeed, in our study we found a number of characteristics that correlated with biological sex. For example, only the females showed increased anxiety after CUMS. This may be related to the prolonged activation of the HPA axis caused by chronic stress and the fact that adrenal volume increases more in females than in males during puberty [32]. On the other hand, high levels of cortisol induced by stress and dysregulation of the HPA axis are known to affect the hippocampus and cause impairments in memory and learning [7]. In our study, we observed sexual dimorphism in hippocampus-dependent forms of memory in the remote period following CUMS. For example, we observed impaired working memory in the passive avoidance test in the females and reduced object memory in the males. In addition, the females showed higher rates of social activity, as well as a decrease in exploratory activity and impaired working memory, in the remote period after CUMS compared to the males. At the same time, decreased motor activity in the remote period after CUMS was characteristic of both sexes. These responses may be due to differential activation of the dopaminergic and serotonergic systems of the brain. For example, the dopaminergic system has been shown to be more sensitive to early-life stressors in men, which increases the likelihood of developing disorders such as attention deficit hyperactivity disorder (ADHD) and schizophrenia. In addition, the brain and neuroendocrine system are sensitive to adrenal hormones during puberty [33], which may be critical when combined with chronic stress.

Various neural structures have been shown to be involved in the maintenance of social memory, including the ventral regions of the hippocampus [25], the medial prefrontal cortex (mPFC), the anterior cingulate cortex (ACC) [34], and entorhinal cortical projections [35]. For example, the mPFC [36] and ACC [37] are implicated in anxiety. One of the aims of this study was to elucidate which biological targets were responsible for the divergent responses of the males and females in the remote period after CUMS. To this end, the hippocampus and cortex were examined separately. Since the females showed increased social engagement and anxiety, accompanied by deficits in memory and learning, it was imperative to first examine both the ventral and dorsal hippocampal morphology. In addition, studying the expression of genes associated with inflammation (specifically IL-6) and SigmaR1 expression was identified as a promising objective, given their potential as targets for neurotropic and antidepressant actions. It has been demonstrated that SigmaR1 agonists facilitate neuronal activity in the hippocampus, thereby replicating the influx of intracellular calcium that is necessary for neuronal excitability and signaling [38]. It was expected that in the males, given their increased exploratory activity, there would be notable changes in the gene expression and morphology of the dorsal hippocampus. Similarly, changes in the ventral hippocampus were expected in the females due to their increased social activity and anxiety. However, no changes were observed. Some studies have reported increases in IL-6 levels in various brain regions [39], including the hippocampus. However, our results did not confirm these findings. In addition, the memory decline and increased anxiety in the females were not correlated with the numbers or shapes of the microglial and astrocytic cells in the hippocampus.

As in [40], our model showed no change in SigmaR1 gene expression in the hippocampus. It is important to note that the housekeeping genes used to assess gene expression may have been suboptimal. As proposed in some studies [41,42], the ActB and B2m genes are unstable. In our model, statistically significant changes in SigmaR1 gene expression after CUMS were observed when normalized using the aforementioned genes. The absence of significant differences in cell morphology, MAP2 staining intensity, and SigmaR1 expression does not support the claim of non-involvement in the stress response. This is due to the fact that SigmaR1 affects physiological processes when it is translocated from the MAM to other cellular compartments [43], in particular the regulation of calcium signaling [44] and ion channels [45]. Therefore, future studies should consider changes in the intracellular localization of SigmaR1 and its interactions with other ion channels. The post-stress phenotype, which is equally expressed in females and males, is characterized by a decrease in locomotor activity. However, studies [46,47] have shown that mice lacking the Sigma-1 receptor exhibit locomotor deficits, muscle weakness, and a loss of motor neurons [46].

MAP2 intensity is commonly used in ischemia studies [29] but may also be an early marker of neuronal damage. Therefore, we analyzed the intensity of MAP2 immunostaining in the entorhinal cortex. No statistically significant results were obtained following CUMS modeling.

For the analysis of the cortex, we examined protein levels by ELISA rather than gene expression. In our model, a statistically significant increase in SigmaR1 was found in brain homogenates after CUMS, independent of biological sex but to a greater extent in males.

SigmaR1 has been shown to promote neuroprotective effects [48,49]. In our model of CUMS, we found a simultaneous decrease in locomotor activity and an increase in the SigmaR1 protein level. However, the analysis of pro-inflammatory cytokines (IL-1β and TNF-alpha) and anti-inflammatory cytokines (IL-10) in the cortical homogenates did not show any statistical differences, except for IL-1β. This interleukin showed a statistically significant decrease in males after CUMS. IL-1β is involved in stress-induced neuroinflammation, which may lead to anxiety-like behavior [50]. In this work, we did not find a decrease in locomotor activity in the males compared to the females, but we did find an increase in cognitive activity, which may have been caused by the lower level of this inflammatory cytokine in the male brain. It is well known that elevated IL-1β levels in the hippocampus correlate with disruption of LTP [51] and inhibition of GABA(A) receptor function, which may critically affect the learning process. It is possible that SigmaR1 plays a neuroprotective role here, with a small degree of sexual dimorphism that can be detected by comparing the control groups. There are data on the neuroprotective role of Sigma-1 receptor ligands in an in vitro model [52] and in a model of motor neuron degeneration. Thus, further studies should focus on the spatial transcriptomics of SigmaR1 since different parts of the brain may respond differently to stress modeling. By analyzing the results of behavioral tests, a morphometric study of the hippocampus, immunoassays of cortical homogenates, and real-time PCR, we found that some of the long-term effects of CUMS are sex-dependent. Among them are anxiety behavior, learning, hippocampus-dependent forms of memory, and the IL-1β concentrations in the cerebral cortices of male and female C57BL/6 mice. At the same time, some effects of CUMS were observed in the animals irrespective of sex. As there are differences in the responses to early-age CUMS, the mechanisms underlying these effects should be further studied taking biological sex into account.

## 4. Materials and Methods

In vivo experiments were carried out in the SPF vivarium of the Central Research Laboratory of PRMU IFM. Female (n = 24) and male (n = 24) mice of the C57BL/6 strain were used in this study. Stress modeling started on postnatal day 17. The animals were maintained in individually ventilated cages at 20 ± 2 °C and 60% humidity, with a 12 h light/dark cycle and constant access to food and water. The maintenance and care of the laboratory rodents was approved by the Bioethics Committee “PRMU” of the Ministry of Health of the Russian Federation. The care and treatment of the animals was in accordance with the “Guide for the Care and Use of Laboratory Animals” (ILAR publication, 1996, National Academy Press) and “Guidelines for the maintenance and care of laboratory animals” (GOST 33216-2014).

CUMS modeling: The scheme of the experiment is shown in Figure 1. CUMS modeling was performed on 12 male mice and 12 female mice from day 17 to day 30 of postnatal development. Detailed descriptions of the stressors are presented in Table 1. On day 31 of postnatal development, the mice were separated from their mothers and placed in new cages with individuals of the same sex and age.

The behavioral tests: The long-term effects of CUMS were studied when the animals reached adulthood at 68–74 days of postnatal development using behavioral tests, as described in Figure 1. The behavioral tests were ordered from the least stressful to the most stressful.

The open-field test: The activity of the males and females was assessed in bright light in a 45 cm × 45 cm × 40 cm square open-field arena (Panlab, Cornella (Barcelona), Spain) divided into two zones (center and periphery). An animal explored the arena for 5 min, and indicators of anxiety, locomotor activity, behavior (grooming, freezing, rearing, and sniffing), and exploratory activity were recorded using a video recording system and Smart Video Tracking System v. 3.0 software (Panlab, Cornella (Barcelona), Spain).

The Crowley test: A study of social behavior was conducted in dim light in an apparatus consisting of a box measuring 60 × 42 × 22 cm that was divided into three chambers by transparent partitions with doors (Panlab, Cornella (Barcelona), Spain). An animal was allowed to move freely between the compartments. Cylinders containing unfamiliar animals of the same strain and sex were placed in the outermost chambers on the left and right. After habituation, the first session was started to assess social activity: an unfamiliar animal was placed in the left cylinder, while the right cylinder remained empty. In the second session, to assess social memory, another unfamiliar animal was placed in the right cylinder, while the animal in the left cylinder was considered already familiar. Each session lasted 5 min with a 10 min break in between. The experimental animal’s transitions between the chambers were recorded by a camera and analyzed using Smart Video Tracking System v. 3.0 software (Panlab, Cornella (Barcelona), Spain). The time spent by the animal interacting with the animals in the cylinders was recorded.

The novel object recognition test: This test was performed in the “Open Field” apparatus (Panlab, Cornella (Barcelona), Spain) in low light. After habituation, an animal was placed in the apparatus to explore two identical objects for 7 min. After an hour, one of the objects was replaced by a new one with a different shape and color. The animal’s activity was recorded by a camera and analyzed using Smart Video Tracking System v. 3.0 software (Panlab, Cornella (Barcelona), Spain). The total time spent on each object was recorded.

The passive avoidance (PA) test: The PA chamber (Panlab, Cornella (Barcelona), Spain) consisted of brightly lit and dark compartments separated by an automatic door, with a floor made of metal bars. During the training phase, an animal was placed in the brightly lit compartment. Then, the animal moved to the dark compartment, where it received an electrical stimulus through the metal bars of the floor (50 Hz and 1.5 mA for 1 s). After 24 h, the test phase was carried out: the animal was returned to the brightly lit compartment, and the latency period of the transition to the dark compartment of the chamber was recorded. Stimulus delivery, the position of the door between the compartments, and the time of the transition from one compartment to the other were recorded automatically using the Shutavoid v1.8.03 program (Panlab, Cornella (Barcelona), Spain).

The Porsolt forced swim test: This test was performed in a glass cylinder that was 40 cm tall, 20 cm in diameter, and filled with water to a depth of 25 cm. The water temperature was +25 °C. During the test, an animal was placed in the cylinder for 5 min. The animal’s strategies, such as climbing, active swimming, and immobility, were recorded by a camera and analyzed using Smart Video Tracking System v. 3.0 software (Panlab, Cornella (Barcelona), Spain). At the end of the test, the mouse was removed from the water and excess water was removed from its fur with a paper towel. The animal was then placed on a heating pad to dry for 15 min before being returned to its home cage.

The animals were then sacrificed, and brain tissues were collected, specifically the cortex and hippocampus for morphological studies, the hippocampus for gene expression studies, and the cortex for quantitative protein analysis.

Immunohistochemistry: Brain tissue from 12 mice was used for immunohistochemical staining. Transcardial perfusion was performed before brain dissection: A heparin solution in 0.1 M PBS was injected for the first 15 s of perfusion. Then, the solution was changed to a 4% PFA solution in 0.1 M PBS with a volume of 50 mL, and the brain was then extracted. Each brain was sectioned along the midline into 2 hemispheres. The left hemisphere was then cut into frontal slices for further analysis of the dorsal hippocampus, while the right hemisphere was cut into horizontal slices for further analysis of the ventral hippocampus and entorhinal cortex. The slices were cut with a total thickness of 300 µm using a McIlwain Tissue Chopper (Loughborough, UK). The prepared sections were fixed in a 4% PFA solution at 4 °C for 24 h. The sections were soaked in an ethylene glycol and sucrose solution to full immersion and stored at −20 °C until immunohistochemical processing. The sections were stained in 48-well plates. The tissues were washed twice to remove the ethylene glycol using a Tris–glycine buffer (0.1 M glycine normalized to pH 7.2–7.4 with a 1 M Tris buffer), followed by incubation in this solution for 1 h on an orbital shaker at 120 rpm. Unspecific binding was then blocked with a mixture of 5% goat serum, 0.5% Triton X-100, and 0.1% Tween 20 in PBS for 1 h at RT using the same shaking characteristics. Each section was then transferred to a 48-well plate containing 300 µL of a mixture of a chicken primary anti-MAP2 antibody (Abcam, Cambridge, UK, AB5392, 1:350), 5% goat serum, and 0.5% Tween 20 in PBS. The plate was kept on an orbital shaker at 50 rpm and 4 °C for 72 h during antibody incubation. The samples were washed three times within 10 min with a wash buffer (0.1% Triton X-100 and 0.1% Tween 20 in PBS) on an orbital shaker at 120 rpm. The sections were then incubated with 400 µL of a mixture of an Alexa Fluor 488 anti-chicken IgY antibody (Invitrogen, Carlsbad, CA, USA, A-11039, 1:500), 5% goat serum, 0.5% Triton X-100, and 0.1% Tween 20 in PBS. Incubation was carried out for 1 h at RT on an orbital shaker at 50 rpm. Following the same procedure, the sections were incubated with a rabbit polyclonal anti-Iba1 antibody (Fujifilm, Tokyo, Japan, 019-19741, 1:500), an Alexa Fluor 555 goat anti-rabbit IgG antibody (Invitrogen, Carlsbad, CA, USA, A-21428, 1:500), a guinea pig anti-GFAP antibody (Synaptic Systems, Goettingen, Germany, 173004, 1:350), and an Alexa Fluor 647 goat anti-guinea pig IgG antibody (Invitrogen, Carlsbad, CA, USA, A-21450, 1:500). After the final wash, the sections were rinsed once with PBS and incubated in 500 µL of a Hoechst 33258 solution (Sigma Aldrich, Burlington, MA, USA, 14530, 1 µg/mL) for 10 min on an orbital shaker at 50 rpm for nuclear visualization. After nuclear staining, the sections were washed with PBS at RT for 10 min on an orbital shaker at 120 rpm and mounted with a DAPI mounting medium (Abcam, Cambridge, UK, ab104139). The final drying of the samples was performed in a refrigerator at 4 °C.

Morphometric analysis: Z-stacks of the dorsal and ventral parts of the hippocampus with a total thickness of 24 µm were prepared to analyze glial cells. As the intensity of MAP2 immunostaining in brain slices can be correlated with the structural dynamics of neuronal networks and dendritic plasticity, a detailed comparative immunofluorescence analysis of the CA1 field in the ventral and dorsal hippocampus was carried out. For this purpose, three randomly selected regions of interest in the CA1 field of the dorsal and ventral hippocampus and the entorhinal cortex were analyzed with the maximum pinhole width (optical thickness = 1 µm). The mean fluorescence intensity was then measured using the General Analysis 3 program (NIS Elements software, Nikon, Tokyo, Japan). The quantitative cell ratio in the 24 µm section was measured using a general image of the stratum radiatum of the CA1 field of the hippocampus. A 2D mask was then created, and parameters such as MinFeret, MaxFeret, area, Circularity, and the shape factor were analyzed. Astrocytes were analyzed using the same binary mask projection scheme. Statistical analysis of data normality was performed using the Kolmogorov–Smirnov test. Group comparison analysis was carried out using the Mann–Whitney test. The data are presented as medians and 25th and 75th percentiles.

ELISA: Brain tissue for ELISA was obtained from 6 mice per group after decapitation. The time from decapitation to freezing the brain tissue samples (hippocampus and cortex) in liquid nitrogen was less than 3 min. The samples were then stored at −80 °C. Prior to analysis, the mouse cortex was thawed, weighed, and homogenized in a hand-held homogenizer using PBS at a ratio of 1:10 compared to the sample’s weight. All procedures were performed on ice. The cortex was centrifuged at 10,000× *g* for 15 min at 4 °C. The supernatant was then quickly frozen in liquid nitrogen. The samples were stored at −80 °C for future use. The TNFa, Il-1b, and Il-10 concentrations were tested using ELISA kits (Cloud-Cone Corporation, Wuhan, China; #SEA133Mu, #MEA563Mu, and #SEA056Mu) according to the manufacturer’s instructions. The ELISA analysis was based on the sandwich method using microtiter plates. The concentrations of MFN1, MARCH5, and SigmaR1 were determined using ELISA kits (BlueGene Biotech, Shanghai, China; #E03M1101, #E03M110, and #E03S0058) according to the manufacturer’s instructions. These kits use a competitive enzyme-linked immunosorbent assay technique with anti-substrate and anti-conjugate antibodies. An APW-200 microplate washer (Allsheng Instruments, Hangzhou, China) was used for automated washing of the plates. The optical density in each well was determined using a multifunctional automated microplate photometer (Synergy™ MXT, BioTek, Winooski, VT, USA) set at 450 nm. A standard curve was generated using software (CurveExpert version 1.4, Hyams Development, Chattanooga, TN, USA), from which the concentration for each sample was interpolated. The Mann–Whitney test was used for statistical analysis, and a *p*-value < 0.05 was considered statistically significant. The data are presented as medians and 25th and 75th percentiles.

Real-time PCR: Total RNA was extracted from the hippocampal tissue using an analogue of TRIzol reagent (Evrogen, Moscow, Russia, BC032) according to the manufacturer’s instructions. Synthesis of cDNA was performed with an MMLV RT kit (Evrogen, SK021S) using random deca-oligonucleotide primers. The PCR reaction was performed using a master mix containing SYBR blue (Biolabmix, Novosibirsk, Russia, MHC030-2040). PCR amplification was performed using a CFX 96 instrument (Bio-Rad, Hercules, CA, USA), with the primer annealing temperature set at 61 °C according to the efficiency value that was previously determined experimentally by the titration method. The following housekeeping genes were used: ActB, Gapdh, Rpl19, B2m, Gusb, and Pum1. The genes of interest were IL-6 and SigmaR1. The primer sequences and efficiency data are provided in Table 2.

Hellemans’ approach was used to evaluate gene expression relative to housekeeping genes [53]. Statistical analysis of data normality was performed using the Kolmogorov–Smirnov criterion. Statistical significance was determined using the non-parametric Kruskal–Wallis criterion. The data are presented as medians and 25th and 75th percentiles.

## Figures and Tables

**Table 1:** Experimental stressors.

Stressor Description	
White noise	White noise (80 dB) was played through a loudspeaker near the home cages for 3 h.
Tilted cage	The Homehome cages were tilted at an angle of 30° for 21 h
Water deprivation	Water deprivation for 9 h
Food deprivation	Food deprivation for 19 h
Overnight illumination	The Mice mice were exposed to normal room light for 21 h.
Foreign object	A Plastic plastic Lego piece was placed into the cage for 21 h.
Shaking	The Mice mice were placed in a plastic box container and shaken orbitally at 100 rpm for 3 h.
Alien sawdust	The Sawdust sawdust in the home cages was replaced with sawdust from other mice for 21 h.
Rat sawdust	Approximately 50 mL of rat sawdust was placed in the cages for 24 h.
Moist sawdust	40 mL of water Water (40 mL) was added to each cage for 5 days.

**Table 2:** List of primers and their characteristics.

Name, 5′→3′, Efficiency (%), Name, 5′→3′, Efficiency (%)					
ACTB_R	GCCGGACTCATCGTACTCC	109	SigmaR F_3	CCTCTTTGGCCAAGACTCCTGA	96
ACTB_F	GTGACGTTGACATCCGTAAAGA		SigmaR _3	GCATGGTATACGCTGCTGTCTGA	
GAPDH_F	CCCACTCTTCCACCTTCGATG	101	IL6_F	GAGACTTCCATCCAGTTGCCTTC	107
GAPDH_R	GTCCACCACCCTGTTGCTGTAG		IL6_R	GAACATGTGTAATTAAGCCTCCGAC	
GusB_F	AGGACGTACTTCAGCTCTGTGAC	114	RPL19 F	TCATCCGCAAGCCTGTACTGT	96
GusB_R	TGCCGAAGTGACTCGTTGCCAA		RPL19 R	ACCTTCTCAGGCATCCGAGCAT	
PUM F	ACAGCCTGCCAACACGTCCTTG	118	B2M F	ACAGTTCCACCCGCCTCACATT	98
PUM R	CCACTGCCAGTGTTGGAGTTTG		B2M R	TAGAAAGACCAGTCCTTGCTGAAG	

## Data Availability

The underlying data were deposited in the Harvard Dataverse repository. Enzyme immunoassay data are available at Shirokova, Olesya; Shchelchkova Natalya; Kozliaeva Elizaveta, 2024, “Data_ELISA_CUMS”, https://doi.org/10.7910/DVN/MKHVYU, Harvard Dataverse, V1. Confocal microscope images are available at Shirokova, Olesya; Korotchenko, Svetlana, 2024, “Images_CUMS”, https://doi.org/10.7910/DVN/8QTL1D, Harvard Dataverse, V1. Real-time PCR data are available at Shirokova, Olesya; Vasilchikov Petr; Pershin Vladimir, 2024, “Real-Time PCR of Mice after CUMS for F1000Research”, https://doi.org/10.7910/DVN/Q4SO0Z, Harvard Dataverse, V1. Data from the behavioral tests are published at Shirokova, Olesya; Daria Kuzmina; Olga Zaborskaya, 2024, “Behavior of mice after CUMS for the F1000 study”, https://doi.org/10.7910/DVN/4F9P6K, Harvard Dataverse, V1.

## Abbreviations

ACC	anterior cingulate cortex
ActB	Actin beta
ADHD	attention deficit hyperactivity disorder
B2m	Beta-2 microglobulin
CA	Cornu Ammonis
cDNA	Complementary deoxyribonucleic acid
CUMS	chronic unpredictable mild stress
C57BL/6	common inbred strain of laboratory mouse
DAPI	4′,6-diamidino-2-phenylindole
ELISA	enzyme-linked immunosorbent assay
ER	endoplasmic reticulum
GABA	Gamma-aminobutyric acid
Gapdh	Glyceraldehyde 3-phosphate dehydrogenase
GFAP	glial fibrillary acidic protein
Gusb	Beta-Glucuronidase
HPA	hypothalamic–pituitary–adrenal
IBA1	Ionized calcium-binding adapter molecule 1
IFM	Institute of Fundamental Medicine
IgG	Immunoglobulin G
IgY	Immunoglobulin Y
IL	interleukin
IP3	Inositol 1,4,5-trisphosphate
LTP	long-term potentiation
MAM	mitochondria-associated ER membrane
MAP2	Microtubule-associated protein 2
MARCH5	membrane-associated ring finger (C3HC4) 5
MFN1	mitofusin-1
MMLV	Murine leukemia virus
mPFC	medial prefrontal cortex
mRNA	Messenger ribonucleic acid
NAc	nucleus accumbens
PA	passive avoidance
PBS	Phosphate-buffered saline
PCR	Polymerase chain reaction
PFA	Paraformaldehyde
PRMU	Privolzhsky Research Medical University
Pum1	Pumilio homolog 1
RNA	ribonucleic acid
Rpl19	Large ribosomal subunit 19
RT	room temperature
SigmaR1	Sigma-1 receptor
SPF	specific-pathogen-free
TNF-α	Tumor necrosis factor
TH	tyrosine hydroxylase
